# Effects of repeated intragastric administrations with heat-inactivated *Mycobacterium aurum* DSM 33539 on the stress-induced aggravation of dextran sulfate sodium (DSS) colitis in C57BL/6N mice

**DOI:** 10.3389/fnins.2024.1488603

**Published:** 2025-01-29

**Authors:** Dominik Langgartner, Anna-Lena J. Weiss, Mattia Amoroso, John D. Sterrett, Christopher A. Lowry, Stefan O. Reber

**Affiliations:** ^1^Laboratory for Molecular Psychosomatics, Department of Psychosomatic Medicine and Psychotherapy, Ulm University Medical Center, Ulm, Germany; ^2^Department of Integrative Physiology, University of Colorado Boulder, Boulder, CO, United States; ^3^Center for Neuroscience, University of Colorado Boulder, Boulder, CO, United States; ^4^Department of Psychology and Neuroscience, University of Colorado Boulder, Boulder, CO, United States; ^5^Center for Microbial Exploration, University of Colorado Boulder, Boulder, CO, United States; ^6^Department of Physical Medicine and Rehabilitation and Center for Neuroscience, University of Colorado Anschutz Medical Campus, Aurora, CO, United States; ^7^German Center for Mental Health (DZPG), Partner Site Mannheim//-Heidelberg//-Ulm, Germany

**Keywords:** *Mycobacterium aurum* DSM 33539, intragastric, chronic subordinate colony housing (CSC), Old Friends, hygiene hypothesis, resilience, inflammation, immunoregulation

## Abstract

Stress-protective effects have been reported for *M. vaccae* NCTC 11659 and *M. vaccae* ATCC 15483^T^. However, it remains to be investigated whether also closely related rapidly growing environmental saprophytic non-tuberculous mycobacteria (NTM) species have protective effects against the negative consequences of chronic psychosocial stress. Therefore, the aim of the current study was to assess whether repeated i.g. administrations of a heat-inactivated preparation of *Mycobacterium aurum* DSM 33539 prior to 19 days of chronic subordinate colony housing (CSC) are able to ameliorate the negative effects of this preclinically validated mouse model for chronic psychosocial stress on subsequent dextran sulfate sodium (DSS) colitis in male C57BL/6N mice. The results of the present study show that repeated i.g. administrations of *M. aurum* DSM 33539 have stabilizing effects on the composition of the gut microbiome, indicated by the findings that *M. aurum* DSM 33539 prevented CSC-induced increases in the relative abundances of the colitogenic phyla Desulfobacterota and Deferribacterota. Indeed, the relative abundance of Deferribacterota on day 19 was strongly correlated with histological damage to the colon. In line with the latter, *M. aurum* DSM 33539 was further protective against the aggravating effects of stress on subsequent DSS colitis. Collectively, our findings confirm and extend previous findings from our group and suggest that the stress-protective effects reported for *M. vaccae* NCTC 11659 and *M. vaccae* ATCC 15483^T^ are generalizable also to other NTM species.

## Introduction

Urbanization is on the rise (United Nations, Department of Economic and Social Affairs, Population Division, 2014), and various stress-associated somatic and mental disorders are more prevalent in urban vs. rural areas of the developed world (Peen et al., 2010; Riedler et al., 2001; Langgartner et al., 2019). Many of these disorders are accompanied by an over-reactive immune system and chronic low-grade inflammation (Pace et al., 2006; Gola et al., 2013), and prospective human and mechanistic animal studies strengthen the idea that an exaggerated immune (re)activity plays a causal role in their pathogenesis (Pace et al., 2006; Hodes et al., 2014; Kivimaki et al., 2014; Rohleder, 2014). Therefore, one possible mechanism predisposing individuals raised in an urban environment, relative to individuals raised in a rural environment, to develop stress-associated disorders, is a long-lasting deficit in immunoregulation, resulting in an uncontrolled/facilitated activation and delayed resolution of the evolutionary-conserved inflammatory stress response. Deficits in immunoregulation are thought to be in part dependent on reduced exposure, especially during early life, to so-called “Old Friends” microorganisms with which mammals co-evolved (Rook, 2013). However, contact with these microorganisms that play a role in setting up regulatory immune pathways is slowly but progressively diminishing due to dramatic changes in global climate, excessive levels of environmental pollution, and recent COVID-19-related restrictions, with urban concrete landscapes of high-income countries being most affected (Rook et al., 2013; Martínez et al., 2015).

Consistent with what is proposed by the “Old Friends” hypothesis, we and others have previously shown that repeated subcutaneous (s.c.) pre-immunizations with a heat-killed preparation of *Mycobacterium vaccae* NCTC (National Collection of Type Cultures) 11659, an abundant saprophytic “Old Friend” from mud with immunoregulatory properties, is effective in: **(i)** stabilizing the gut microbiome (Reber et al., 2016a; Foxx et al., 2020), **(ii)** increasing the percentage of Tregs in mesenteric lymph node cells (Reber et al., 2016a), **(iii)** preventing stress-induced colitis and proinflammatory cytokine secretion from freshly isolated mesenteric lymph node cells stimulated with anti-CD3 antibody *ex vivo* (Reber et al., 2016a), **(iv)** preventing stress-induced aggravation of dextran sulfate sodium (DSS)-induced colitis (Reber et al., 2016a), **(v)** preventing stress-induced exaggeration of anxiety (Reber et al., 2016a), **(vi)** preventing stress-induced microglial priming and neuroinflammation (Frank et al., 2019, 2018; Fonken et al., 2018a,b), **(vii)** ameliorating features of age-associated microglia activation in the amygdala and hippocampus (Sanchez et al., 2022), **(viii)** preventing negative outcomes of sleep deprivation (Bowers et al., 2020), and **(ix)** enhancing fear extinction (Hassell et al., 2019). In an extension of these findings and in support of using “Old Friends” not only to prevent but also to treat stress-associated disorders, we recently showed that *M. vaccae* NCTC 11659 also ameliorates stress-induced anxiety when administered repeatedly via the s.c. route during chronic psychosocial stressor exposure, i.e., after the first psychosocial traumatization has occurred (Amoroso et al., 2020). Finally, we showed in male mice that i.g. *M. vaccae* NCTC 11659 administrations protect against: **(i)** the stress-induced increase in splenic TLR2^+^ and TLR4^+^ polymorphonuclear myeloid-derived suppressor cells (PMN-MDSCs) and TLR4^+^ monocytes/mononuclear (MO)-MDSCs; **(ii)** the increase in functional splenic *in vitro* glucocorticoid (GC) resistance typically seen following psychosocial stress in combination with significant wounding; and **(iii)** the stress-induced increase in basal and LPS-induced splenic *in vitro* cell viability (Langgartner et al., 2023). Our recent findings confirm the protective effects of *M. vaccae* NCTC 11659 against stress-induced aggravation of DSS colitis even when administered via the non-invasive intranasal (i.n.) route prior to or during stress exposure, respectively (Amoroso et al., 2019).

Although our earlier findings already show that the stress-protective effects of *M. vaccae* NCTC 11659 are not specific for this strain but transferable also to *M. vaccae* ATCC (American Type Culture Collection) 15483^T^ (Loupy et al., 2021), it remains to be investigated whether closely related rapidly growing environmental saprophytic non-tuberculous mycobacteria (NTM) species have protective effects against the negative consequences of chronic psychosocial stress. Therefore, the aim of the current study was to assess whether repeated i.g. administrations of a heat-inactivated preparation of *Mycobacterium aurum* DSM 33539 prior to 19 days of chronic subordinate colony housing (CSC) are able to ameliorate the negative effects of this preclinically validated mouse model for chronic psychosocial stress on subsequent DSS colitis in male C57BL/6N mice. The CSC model is based on chronic subordination (19 days) of four male mice toward a dominant male conspecific (Reber et al., 2016a; Langgartner et al., 2015). Compared to respective single-housed control (SHC) mice, CSC is known to, among other physiological and behavioral effects, induce an anxiety-like phenotype (Amoroso et al., 2020; Langgartner et al., 2017; Slattery et al., 2012) as well as to aggravate DSS-induced colitis (Reber et al., 2016a; Amoroso et al., 2019; Reber et al., 2008) and promote the development of spontaneous colitis (Reber et al., 2016a; Langgartner et al., 2017; Reber et al., 2007, 2011).

## Materials and methods

### Animals

Male C57BL/6N mice weighing 17–19 g (~5 weeks of age) were used as experimental mice and male CD-1 mice (Charles River, Sulzfeld, Germany) weighing 30–35 g were used as dominant aggressor mice. Standard polycarbonate mouse cages (16 cm width × 22 cm length × 14 cm height) were used. All mice were kept in a specific pathogen-free (SPF) animal facility under standard laboratory conditions (12-h light–dark cycle, lights on at 06:00 a.m., 22°C, 60% humidity) and had free access to tap water and standard mouse diet. Reporting of the animal study was carried out in accordance with the ARRIVE guidelines 2.0 (Percie du Sert et al., 2020), and the study was approved by the Federal Animal Care and Use Committee (Regierungspräsidium Tübingen, Germany). All efforts were made to minimize the number of animals used and their suffering. Given that the CSC paradigm is based on territorial aggression and the establishment of a social hierarchy, only male mice were used in the present study.

### Experimental procedures

**Figure 1:**
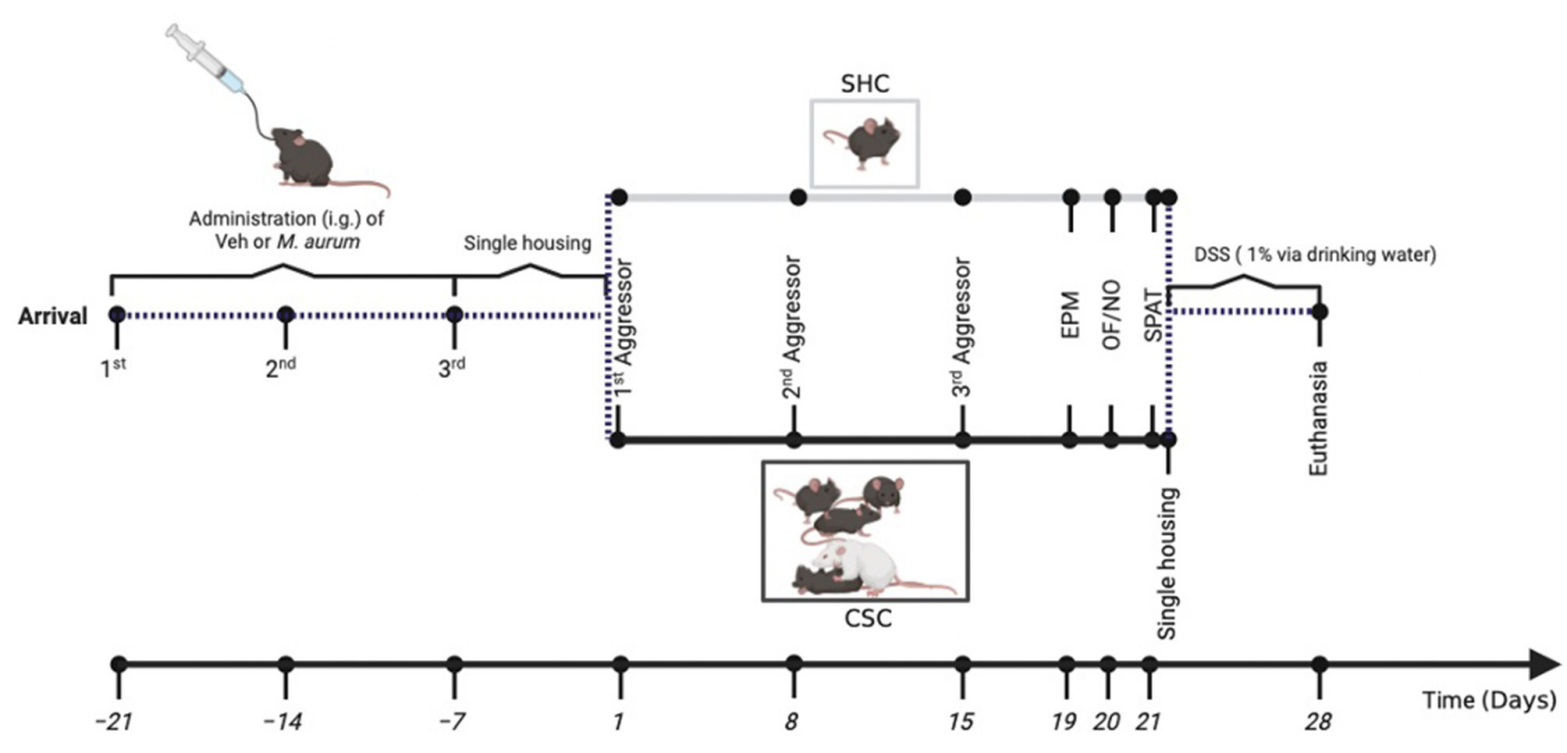
Experimental timeline: male C57BL/6N mice received repeated i.g. administrations of *M. aurum* DSM 33539 or sterile water (vehicle, Veh) on days -21, -14, and -7, before being individually housed for 1 week and subsequently exposed to either 21 days of chronic subordinate colony housing (CSC) or single-housed control (SHC) conditions. Stool samples for the assessment of the fecal microbiome were collected on days 1 and 19. General anxiety was assessed employing the elevated plus-maze (EPM; day 19) and the open-field/novel object (OF/NO; day 20) test, followed by an assessment of social anxiety employing the social preference/avoidance test (SPAT; day 21). Immediately after the SPAT, on day 21, all experimental mice were housed singly and received 1% dextran sulfate sodium (DSS) via their drinking water for seven consecutive days (days 21–28). All mice were euthanized on the morning of day 28 for assessment of physiological and immunological parameters. Figure was created with BioRender.com.

A diagrammatic illustration of the experimental timeline is shown in Figure 1. Following arrival on day -21, all C57BL/6N mice were housed in groups of four and received repeated i.g. administrations of either heat-killed *Mycobacterium aurum* DSM 33539 *(n* = 17) or its vehicle (Veh) sterile water (*n* = 16) on days -21, -14, and -7. Mice were housed individually following the last administration on day -7 until the start of the CSC paradigm on day 1 or remained single-housed (SHC) for control (Veh-SHC: *n* = 8; Veh-CSC: *n* = 8; *M. aurum*-SHC: *n* = 9; *M. aurum*-CSC: *n* = 8). Fecal pellets for compositional analysis of the intestinal microbiome were collected at different experimental days before as well as during the CSC paradigm. Behavioral tests [i.e., elevated plus-maze (EPM, day 19), open-field/novel object test (OF/NO, day 20), and social preference/avoidance test (SPAT, day 21)] were performed to assess changes in general and social anxiety-related behaviors. After the SPAT, all mice were single housed and received 1% dextran sulfate sodium (DSS; MP Biomedicals, Santa Ana, CA, USA) in their drinking water for 1 week. On day 28, mice were euthanized between 07.00 and 10.00 a.m. by decapitation following brief CO_2_ inhalation for assessment of adrenal weight, *ex vivo* interferon gamma (IFN-γ) secretion from anti-CD3/CD28-stimulated mesenteric lymph node cells (mesLNCs), colon length, and histological damage score of the colon.

### Intragastric administration of *Mycobacterium aurum* DSM 33539

A whole heat-killed preparation of *M. aurum* DSM 33539 [supplied as 20 mg/ml of stock solution; provided by Aurum Switzerland AG, Alfred-Escher-Strasse 26, 8002 Zurich, Switzerland (Nouioui and Dye, 2021)] was administered intragastrically (i.g.) via a 20-gauge gavage needle on days -21, -14, and -7 prior to CSC or SHC conditions. *M. aurum* DSM 33539 was diluted in sterile water (Ampuva) at a final concentration of 1 mg/ml, and each mouse received 100 μl of *M. aurum* DSM 33539, equivalent to a final dose of 0.1 mg *M. aurum* DSM 33539/mouse. Of note, this is the same dose that our group has used previously for s.c. and i.g. administration of *M. vaccae* NCTC 11695 and *M. vaccae* ATCC 15483 in mice (Reber et al., 2016a; Amoroso et al., 2020; Langgartner et al., 2023; Amoroso et al., 2019; Loupy et al., 2021).

### Chronic subordinate colony housing paradigm

The CSC paradigm was performed as described previously (Langgartner et al., 2015; Reber et al., 2008, 2007; Foertsch and Reber, 2020). Briefly, mice were weighed and assigned to either the SHC or the CSC group, according to their body weight. To induce chronic psychosocial stress, four CSC mice were housed together with a dominant male CD-1 aggressor mouse for 21 consecutive days. To avoid habituation, CSC mice were transferred to the home cages of novel dominant aggressor CD-1 mice on days 8 and 15. Before the beginning of the CSC procedure, all potential dominant CD-1 mice were tested for their aggressiveness to exclude individuals severely injuring their conspecifics. SHC mice remained undisturbed in their home cages except for changing their bedding one time a week. In a previous study, we convincingly demonstrated that single housing is the adequate control group for the CSC paradigm, as unfamiliar group-housed males also establish a social hierarchy, negatively affecting parameters assessed routinely in studies employing the CSC paradigm (Singewald et al., 2009).

### Analysis of general and social anxiety-related behavior

For assessment of general and social anxiety-related behavior, experimental mice were exposed to the EPM, OF/NO, and SPAT on days 19–21, respectively, as previously described (Amoroso et al., 2020).

#### Elevated plus-maze

To assess treatment (*M. aurum* DSM 33539 and/or CSC) effects on anxiety-related behavior, the EPM test was performed on day 19 of the CSC paradigm as previously described (Reber et al., 2007). Mice were tested for 5 min between 07.00 and 10.00 a.m. The test took place in a dedicated behavioral box inside the animal room where all animals were housed for the whole duration of the experiment. Briefly, the plus-maze consists of two open (length: 30 cm; width: 6 cm; 140 lx) and two closed (length: 30 cm; width: 6 cm; height: 17 cm, 85 lx) arms radiating from a central platform (6 × 6 cm) to form a plus-shaped figure elevated 130 cm above the floor. The open arm edges were 0.3 cm in height to avoid falling. Each mouse was placed in one of the closed arms for 30 s to habituate before the test started. During this time, the entrance to the other arms of the maze was blocked. The maze was thoroughly cleaned with water after each test. In order to assess the number of entries into the closed arms, as well as the relative time spent in the open arms (percentage of time spent in the open arms relative to the amount of time spent in all arms), each test was videotaped and scored afterward by an observer blind to the treatment using a video/computer setup.

#### Open-field/novel object test

To further assess general anxiety-related behavior, the OF/NO test was conducted on day 20 between 07:00 and 10:00 a.m. of CSC exposure as previously described (Amoroso et al., 2020, 2019; Langgartner et al., 2017). Briefly, during OF exposure, the test arena (45 cm length × 27 cm width × 27 cm height; 350 lux) was subdivided into an inner (9 × 27 cm) and an outer zone. The mouse was placed into the inner zone and allowed to explore the arena for 5 min. After 5 min of OF exploration, a round plastic object (diameter: 3.5 cm; height: 1.5 cm) was placed in the middle of the arena and the mouse was allowed to explore the arena containing the unfamiliar NO for 5 min. During OF exploration, the overall distance moved, the time in the inner zone, and the time in the corners were assessed. During NO exploration, the overall distance moved, the time spent in direct contact with the NO, and the time in the corners was analyzed. The test arena was cleaned thoroughly with water before each test. All parameters were analyzed using EthoVision XT (v11.5.1022; Noldus Information Technology, Wageningen, The Netherlands).

#### Social preference/avoidance test

To assess general and social anxiety-related behavior, the SPAT was conducted on day 21 between 07:00 and 10:00 a.m. as described previously (Amoroso et al., 2020, 2019; Slattery et al., 2012). Briefly, the experimental mouse was placed in the SPAT box (45 cm length × 27 cm width × 27 cm height; 20 lux) for 30 s to habituate to the unfamiliar environment before a small empty wire mesh cage (8.5 cm length × 7.5 cm width x 6.5 cm height) was introduced for 150 s. This initial period informs about general anxiety-related behavior as the mouse is exposed to a novel object. Afterward, the empty cage was exchanged with an identical cage containing an unfamiliar male CD-1 mouse for another 150 s. Total distance moved and the entries into the cage zone were recorded using EthoVision XT (v11.5.1022; Noldus Information Technology) during both 150-s trials. The box was cleaned thoroughly with water before every test.

### Dextran sulfate sodium treatment

Immediately after the SPAT on day 21, all experimental mice were housed singly and received 1% DSS (50 kDa; Cat. no. 16011036, MP Biomedicals) through their drinking water for seven consecutive days (days 21–28). The DSS bottles were weighed at the beginning and at the end of the treatment and the amount of DSS consumed was calculated. Given that drinking behavior and, thus, overall DSS consumption varied between the groups all physiological and colitis-related data were corrected by the amount of DSS powder (g) consumed.

### Collection of adrenal glands

On the morning of day 28 mice were decapitated following brief CO_2_ inhalation. Afterward, adrenals were removed, pruned of fat, and weighed.

### Isolation and incubation of mesenteric lymph node cells

To assess treatment (*M. aurum* DSM 33539 and CSC) effects on *ex vivo* anti-CD3/CD28-stimulated cytokine secretion from isolated mesLNCs, mesenteric lymph nodes were removed and stored in ice-cold Roswell Park Memorial Institute Medium (RPMI-1640, Cat. no. R8758, Sigma-Aldrich, St. Louis, MO, USA) supplemented with 10% fetal bovine serum (FBS, Cat. no. 10270106, Gibco^®^, Thermo Fisher Scientific, Waltham, MA, USA), 100 U/mL penicillin and 100 μg/mL streptomycin (Cat. no. 15070063, Gibco^®^, Thermo Fisher Scientific), and 3 × 10^−5^ M β-mercaptoethanol (Cat. no. M6250, Sigma-Aldrich). Lymph nodes were mechanically disrupted and filtered through a cell strainer (Corning™, 70-μm nylon, Cat. no. 431751, Thermo Fisher Scientific). Afterward, cells were washed in cell culture medium and adjusted to a concentration of 2 × 10^5^ cells/100 μl. Twenty-four hours before lymph node cell incubation, wells of a 96-well plate were pre-coated with 100 μl of anti-CD3 antibody (Cat. no. MA1-10184, Thermo Fisher Scientific) diluted in PBS (final concentration: 2.5 μg/ml/well). The wells were then aspirated and washed two times with PBS before lymph node cells were transferred to the wells. 2 × 10^5^ cells were plated per well. Following plating, 100 μl anti-CD28 antibody (Cat. no. 16-0281, e-Bioscience™, Thermo Fisher Scientific) diluted in RPMI were added to the wells at a final concentration of 1 μg/ml/well. After 48-h incubation (37°C, 5% CO_2_), supernatants were removed and stored at −20°C until interferon-gamma (IFN-γ) concentrations were measured in the supernatants using a commercially available ELISA kit.

### Enzyme-linked immunosorbent assay for assessment of *ex vivo* IFN-γ secretion from anti-CD3/CD28-stimulated mesLNCs

Interferon-γ concentration in the supernatants of *ex vivo* anti-CD3/CD28-stimulated mesLNCs was measured using a commercially available ELISA kit according to the manufacturer's protocol (Cat. no. DY485-05, DuoSet ELISA, R&D Systems Europe, Ltd., Abingdon, UK).

### Determination of colon length and histological damage score of the colon

To assess CSC effects on intestinal inflammation, the colon was removed. After its length was measured, it was mechanically cleaned of feces. To prepare histological sections, 1 cm of the distal third of the colon was cut longitudinally and fixed in 4% formalin overnight. The next day, the fixed tissue was embedded in paraffin and cut longitudinally. Histological slides were obtained from the paraffin blocks, stained with hematoxylin–eosin, and evaluated by histological scoring performed by an investigator blinded to treatment. Histological damage score was assessed as previously described (Reber et al., 2008): epithelium score (0: normal morphology; 1: loss of goblet cells (i.e., cells characterized by large mucin granules); 2: loss of goblet cells in large areas; 3: loss of crypts; 4: loss of crypts in large areas) and infiltration score (0: no infiltration; 1: infiltrate around crypt bases; 2: infiltrate reaching to lamina muscularis mucosae; 3: extensive infiltration reaching the lamina muscularis mucosae and thickening of the mucosa with abundant edema; 4: infiltration of the lamina submucosa). The total histological score of each mouse represents the sum of the epithelium and infiltration scores and ranges from 0 to 8.

### Ultra-high-throughput microbial community analysis on the illumina sequencing platform

For collection of fecal samples, mice were placed in a clean cage without bedding until defecating three normal-sized fecal pellets. After mice were placed back into their home cage, fecal samples were collected with a sterile G21 syringe needle. Fecal samples for analysis of the diversity and community composition of the fecal microbiome were collected before (day 1) and at the end of the CSC paradigm (day 19), respectively, and frozen at −80°C until DNA extraction was performed. DNA extraction was performed as previously described with minor modifications (Appiah et al., 2021). Briefly, DNA was extracted using the PowerSoil DNA Extraction Kit (MoBio Laboratories, Carlsbad, CA, USA) according to the manufacturer's instructions. Samples were analyzed using 16S rRNA gene amplicon sequencing. Therefore, marker genes in isolated DNA were PCR-amplified using GoTaq Master Mix (Promega, WI, USA) and a primer pair (Integrated DNA Technologies, Coralville, IA, USA) targeting the V4 hypervariable region of the 16S rRNA gene modified with a unique 12-base sequence identifier for each sample and the Illumina adapter. The thermal cycling program consisted of an initial step at 94°C for 3 min followed by 35 cycles (94°C for 45 s, 50°C for 1 min, and 72°C for 1.5 min), and a final extension at 72°C for 10 min. PCRs were run in duplicate, and the products from the duplicate reactions were pooled and visualized on an agarose gel to ensure successful amplification. PCR products were cleaned and normalized using the SequalPrep Normalization Kit (Thermo Fisher Scientific) following the manufacturer's instructions. After measuring the correct size distribution of the normalized library on a DNA1000 ScreenTape^®^ using a Tape Station 4200 (Agilent), the amplicon pool was sequenced on an Illumina MiSeq using V2 chemistry and 2 × 250 bp paired-end sequencing at the Core Facility Genomics (Medical Faculty at Ulm University). Bioinformatic analysis was conducted in close collaboration with the Core Facility for Bioinformatics and Data Management run by the Medical Faculty at Ulm University. Briefly, microbiome analysis was performed using the Quantitative Insights Into Microbial Ecology program (QIIME2-2021.4; http://qiime2.org) (Bolyen et al., 2019). Raw sequence data were demultiplexed and quality filtered using the q2-demux plugin followed by denoising with DADA2 via q2-dada2 (Callahan et al., 2016) to identify all observed amplicon sequence variants (ASVs) [i.e., 100% operational taxonomic units (OTUs)]. In DADA2, forward reads were trimmed at position 19 and truncated at position 151; reverse reads were trimmed at position 19 and truncated at position 151, based on sequence quality. Taxonomy assignment was conducted using a naïve Bayes classifier trained on the SILVA version 128 database (Quast et al., 2013). A phylogenetic tree was created using the SATé-enabled phylogenetic placement (SEPP) fragment insertion method via QIIME2 (Janssen et al., 2018). Alpha diversity analyses were executed within QIIME2, with data rarefied to an even sampling depth of 1,500 reads per sample (Weiss et al., 2017). Alpha-diversity metrics [i.e., Pielou's evenness, Shannon diversity index, Faith's phylogenetic diversity, observed ASVs were evaluated after samples were rarefied. Code for reproducing microbiome analysis can be found at https://github.com/GRalexOSS/Maurum_Processing].

### Statistics

For statistical analysis and graphical illustrations, GraphPad Prism (version 10, GraphPad Software, LCC) was used. Statistical analysis was performed as previously described (Kempter et al., 2021, 2023). Briefly, Kolmogorov–Smirnov test with Lilliefors' correction was employed to test for normal distribution. Extreme outliers in normally distributed data sets were identified by the Grubbs test (Grubbs, 1969) and excluded from further analysis. Normally distributed data sets were analyzed by parametric statistics, i.e., two-tailed Student's *t*-test (one factor, two independent samples), two-tailed Student's *t*-tests with Welch's correction when appropriate, and two-way ANOVA (two factors, two or more independent samples). In case of repeated measures, data were analyzed using a linear mixed model (LMM). Non-normally distributed data sets were analyzed by non-parametric statistics, i.e., Mann–Whitney *U*-test (one factor, two independent samples) and Wilcoxon test (one factor, two dependent samples). All statistical tests comparing more than two samples were followed by *post hoc* analysis using Bonferroni pairwise comparison when a significant main effect was found. Data are presented as bars (mean + SEM) with individual values. The two-tailed level of significance was set at *P* ≤ 0.05.

## Results

### Effects of *M. aurum* DSM 33539 on general/social anxiety

#### Elevated plus-maze

Statistical analysis revealed that *M. aurum* DSM 33539 vs. Veh mice of the SHC group showed a significantly reduced number of entries into the closed arms [Table 1; Factor *M. aurum* DSM 33539 × Factor Stress Interaction: *F*_(1, 29)_ = 7.168, *P* = 0.012; Bonferroni: *P* = 0.029] and percentage of time spent in the open arms [Table 1; Factor *M. aurum* DSM 33539 × Factor Stress Interaction: *F*_(1, 28)_ = 8.172, *P* = 0.008; Bonferroni: *P* = 0.006].

**Table 1:** EPM, OF/NO, and SPAT behavioral tests.

	**Veh**		***M. aurum***	
	**SHC**	**CSC**	**SHC**	**CSC**
**EPM**				
Entries to closed arms (*n*)	15.00 ± 1.16	10.88 ± 1.62	9.78 ± 1.48^#^	13.38 ± 1.44
Percentage time spent in open arms (%)	10.63 ± 2.95	6.19 ± 1.11	3.67 ± 0.93^##^	8.11 ± 1.17
**OF/NO**				
Distance moved (OF) (cm)	1,889.24 ± 73.51	1,615.38 ± 161.47	1,771.01 ± 141.86	1,495.81 ± 86.23^*^
Time in inner zone (OF) (s)	24.89 ± 4.67	40.12 ± 13.43	28.54 ± 4.67	24.68 ± 8.38
Time in corners (OF) (s)	139.81 ± 13.54	145.36 ± 24.52	162.28 ± 13.33	175.74 ± 14.88
Distance moved (NO) (cm)	1,841.82 ± 145.72	1,526.14 ± 103.52	2,090.53 ± 141.61	1,353.46 ± 96.80^***^
Time in contact zone (NO) (s)	10.87 ± 2.23	12.73 ± 2.77	13.50 ± 2.81	6.77 ± 1.69
Time in corners (NO) (s)	135.44 ± 18.43	150.59 ± 22.25	139.33 ± 13.70	206.42 ± 14.30^*^
**SPAT**				
Distance moved empty cage compartment (cm)	504.63 ± 67.06	389.20 ± 54.52	548.93 ± 88.78	418.41 ± 75.54
Distance moved social cage compartment (cm)	724.28 ± 73.16	525.28 ± 67.95	664.61 ± 74.20	452.76 ± 39.11
Entries into cage zone during empty cage exploration (s)	2.38 ± 0.63	2.00 ± 0.50	2.33 ± 0.76	1.63 ± 0.56
Entries into cage zone during social cage exploration (s)	4.75 ± 0.82^§^	2.88 ± 0.69	3.56 ± 0.60	2.50 ± 0.42

CSC, chronic subordinate colony housing; EPM, elevated plus-maze; *M. aurum, Mycobacterium aurum* DSM 33539; OF/NO, open-field/novel object; SHC, single-housed control; SPAT, social preference/avoidance test; Veh, vehicle (sterile water).

^*^P ≤ 0.05, ^***^P ≤ 0.001 vs. respective SHC.

^#^*P* ≤ 0.05, ^##^*P* ≤ 0.01 vs. respective Veh group.

^§^*P* ≤ 0.05 vs. empty cage condition.

#### Open-field/novel object test

The distance moved during OF (Table 1; MWU; *P* = 0.036) and NO (Table 1; Factor Stress: *F*_(1, 29)_ = 17.59, *P* < 0.001; Bonferroni: *P* < 0.001) exploration was significantly reduced, while the time in the corners during NO exploration (Table 1; Factor Stress: *F*_(1, 29)_ = 5.624, *P* = 0.025; Bonferroni: *P* = 0.019) was significantly increased in CSC vs. SHC mice of the *M. aurum* DSM 33539 group. The time in the inner zone and the time in corners, both during OF exploration and the time in the contact zone during NO exploration, were neither affected by CSC nor *M. aurum* DSM 33539.

#### Social preference/avoidance test

Statistical analysis revealed a significant main effect of Factor Social [*F*_(1, 29)_ = 10.71, *P* = 0.003] and Factor Stress [*F*_(1, 29)_ = 7.880, *P* = 0.009] in the distance moved during SPAT (Table 1). Moreover, SHC mice of the Veh groups showed significantly more entries into the cage zone during social vs. empty cage exploration [Factor Social *F*_(1, 29)_ = 12.62, *P* = 0.001; Bonferroni: *P* = 0.050].

### Effects of *M. aurum* DSM 33539 on CSC-induced adrenal enlargement and aggravation of DSS-induced colitis

**Figure 2:**
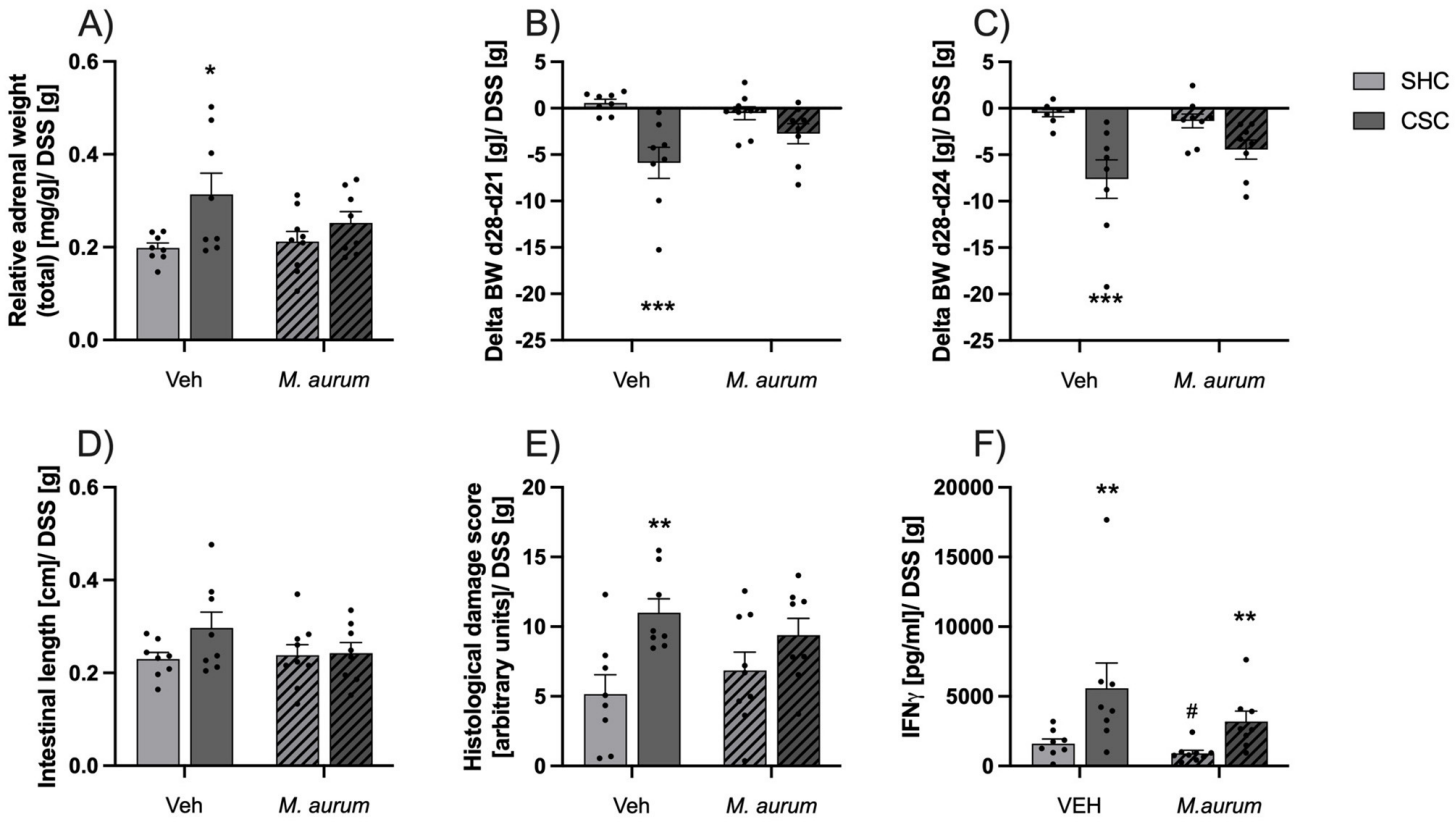
*M. aurum* DSM 33539 protects against CSC-induced adrenal enlargement and aggravation of DSS-induced colitis. **(A)** Relative adrenal weight, **(B)** delta body weight between days (d)28-21, **(C)** delta body weight between d28-24, **(D)** intestinal length, **(E)** histological damage score of the colon, and **(F)** interferon (IFN)-γ secretion from isolated and anti-CD3/CD28-stimulated mesenteric lymph node cells (mesLNCs). Given that drinking behavior and, thus, overall DSS consumption varied between the groups all physiological and colitis-related data were corrected by the amount of DSS powder (g) consumed. CSC, chronic subordinate colony housing; *M. aurum, Mycobacterium aurum* DSM 33539; SHC, single-housed control; Veh, vehicle (sterile water). Data are presented as mean + SEM including individual values. *P ≤ 0.05, **P ≤ 0.01, ***P ≤ 0.001 vs. respective SHC condition; ^#^*P* ≤ 0.05 vs. respective Veh condition.

Relative adrenal weight was significantly increased in CSC vs. SHC mice of the Veh but not *M. aurum* DSM 33539 group [Figure 2A; two-way ANOVA: Factor Stress: *F*_(1, 29)_ = 7.621, *P* = 0.010; Bonferroni: *P* = 0.016]. Moreover, CSC vs. SHC mice of the Veh but not *M. aurum* DSM 33539 group showed an increased body weight loss during DSS treatment between days 21 and 28 (Figure 2B, MWU: *P* < 0.001) and between days 24 and 28 [Figure 2C, two-way ANOVA: Factor Stress: *F*_(1, 29)_ = 17.84, *P* < 0.001; Bonferroni: *P* < 0.001], as well as an increased histological damage score (Figure 2E; MWU; *P* = 0.005), while colonic length was comparable between all groups (Figure 2D). *Ex vivo* IFN-γ secretion from anti-CD3/CD28-stimulated mesLNCs was increased in CSC vs. SHC mice of both the Veh and *M. aurum* DSM 33539 group (Figure 2F; MWU; Veh: *P* = 0.01; *M. aurum*: *P* = 0.002) with *M. aurum* DSM 33539 vs. Veh mice of the SHC group secreting significantly less IFN-γ (MWU; *P* = 0.046).

### Effects of *M. aurum* DSM 33539 on α-diversity of the fecal microbiome

**Figure 3:**
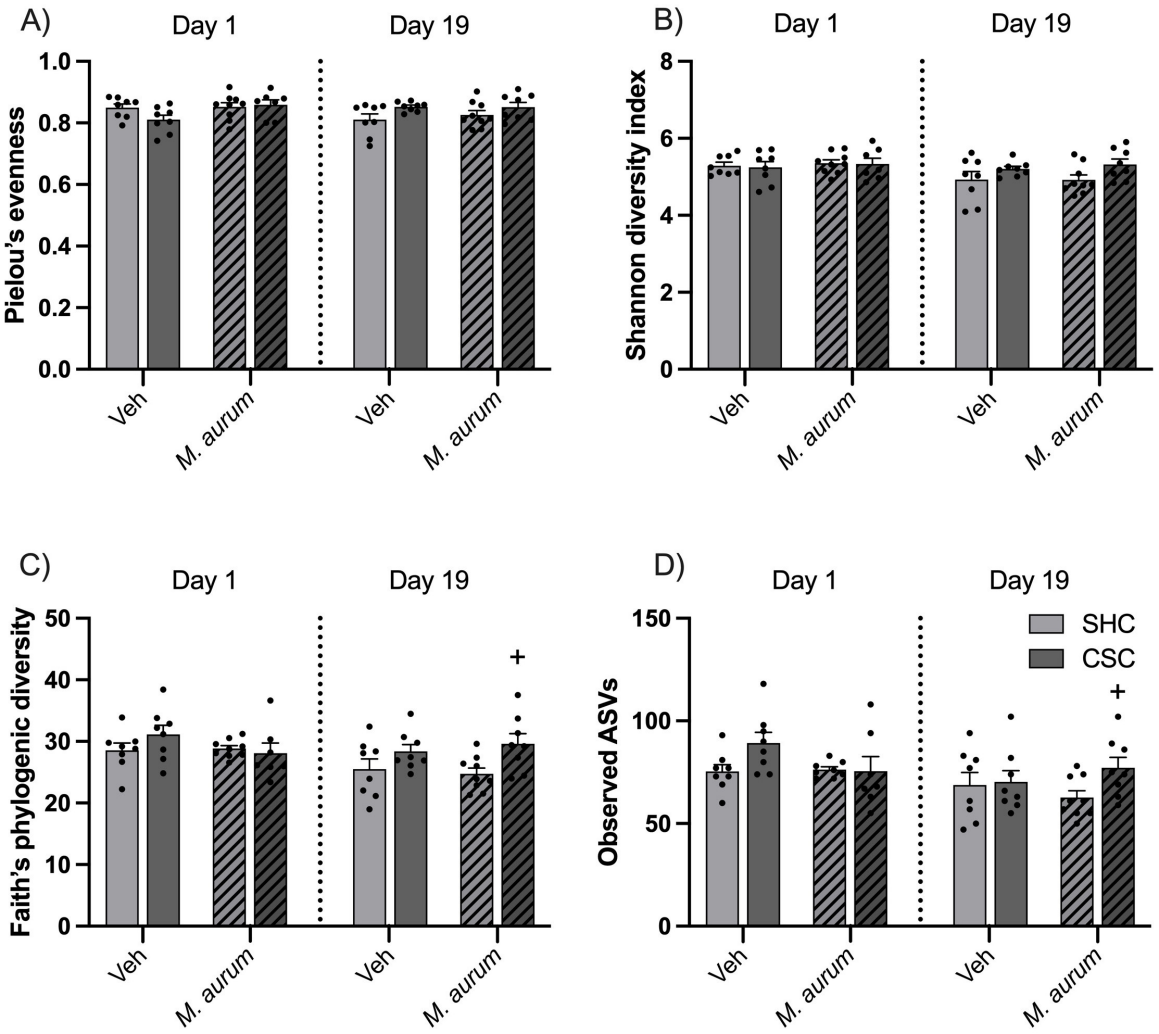
Effects of *M. aurum* DSM 33539 on alpha diversity measures. Alpha diversity metrics **(A)** Pielou's evenness, **(B)** Shannon diversity index, **(C)** Faith's phylogenetic diversity (PD), and **(D)** observed amplicon sequence variants (ASVs). CSC, chronic subordinate colony housing; *M. aurum, Mycobacterium aurum* DSM 33539; SHC, single-housed control; Veh, vehicle (sterile water). Data are presented as mean + SEM including individual values. ^+^*P* ≤ 0.05 vs. respective SHC condition, analyzed via separate Student's *t*-test.

Statistical analysis revealed a significant interaction effect for Pielou's evenness [Figure 3A; LMM: Factor Time × Factor Stress Interaction: *F*_(1, 28)_ = 8.349, *P* = 0.007], a significant main effect of time for Shannon diversity index [Figure 3B; LMM: Factor Time: *F*_(1, 28)_ = 5.264, *P* = 0.029], a significant main effect of time [Figure 3C; LMM: Factor Time: *F*_(1, 28)_ = 5.711, *P* = 0.024] and stress [Figure 3C; LMM: Factor Stress: *F*_(1, 29)_ = 6.447, *P* = 0.017] for Faith's phylogenetic diversity and a significant main effect of time [Figure 3D; LMM: Factor Time: *F*_(1, 27)_ = 8.640, *P* = 0.007] and stress [Figure 3D; LMM: Factor Stress: *F*_(1, 29)_ = 4.196, *P* = 0.050] as well as an interaction effect [Figure 3D; LMM: Factor *M. aurum* DSM 33539 × Factor Time × Factor Stress Interaction: *F*_(1, 27)_ = 4.517, *P* = 0.043] for observed ASVs, respectively. Although Bonferroni *post hoc* analysis did not reveal significant differences for any of the four α-diversity metrics, analysis via separate Student's *t*-tests between SHC and CSC mice of the *M. aurum* DSM 33539 group at day 19 revealed a by-trend increase in Shannon index (*P* = 0.053; Figure 3B), and a significant increase in Faith's phylogenetic diversity (*P* = 0.017; Figure 3C) and observed ASVs (*P* = 0.028; Figure 3D).

### Effects of *M. aurum* DSM 33539 on the community composition of the fecal microbiome

**Figure 4:**
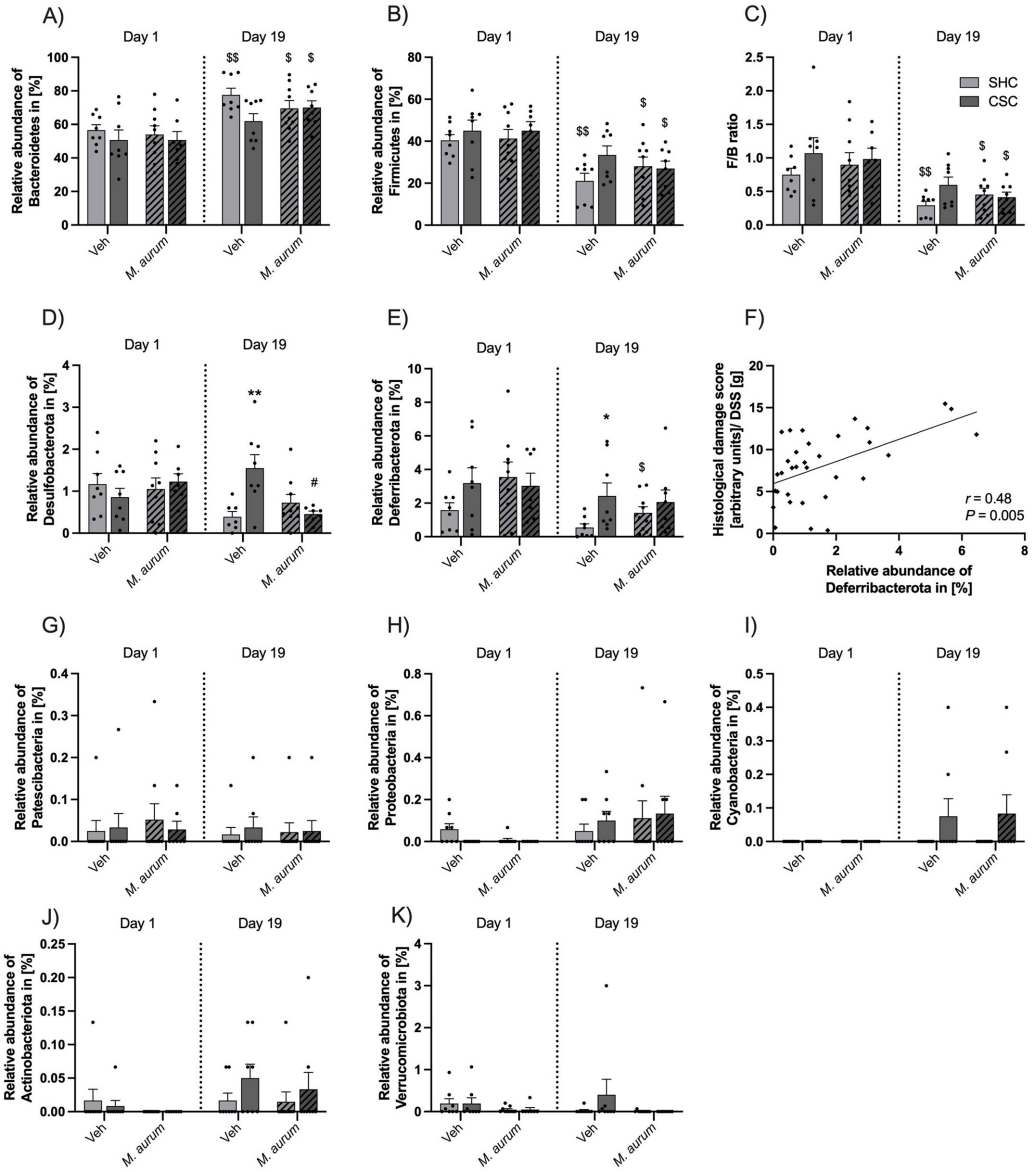
Effects of *M. aurum* DSM 33539 on community composition of the gut microbiome. Relative abundance of **(A)** Bacteroidetes and **(B)** Firmicutes. **(C)** Firmicutes/Bacteroidetes ratio. Relative abundance of **(D)** Desulfobacterota, **(E)** Deferribacterota, **(F)** Spearman correlation analysis between the relative abundance of Deferribacterota in (%) and the histological damage score of the colon corrected by the amount of DSS powder (g) consumed, **(G)** Patescibacteria, **(H)** Proteobacteria, **(I)** Cyanobacteria, **(J)** Actinobacteriota, **(K)** Verrucomicrobiota. CSC, chronic subordinate colony housing; *M. aurum, Mycobacterium aurum* DSM 33539; SHC, single-housed control; Veh, vehicle (sterile water). Data are presented as mean + SEM including individual values. *P ≤ 0.05, **P ≤ 0.01 vs. respective SHC condition; ^$^*P* ≤ 0.05, ^$$^*P* ≤ 0.01 vs. respective day 1; ^#^*P* ≤ 0.05 vs. respective Veh condition.

Analysis of the fecal microbiome composition revealed a significant increase in the relative abundance of Bacteroidetes (Figure 4A) and a significant reduction in the relative abundance of Firmicutes (Figure 4B) in SHC mice of the Veh group (Bacteroidetes, Wilcoxon: *P* = 0.008; Firmicutes, Wilcoxon: *P* = 0.008) as well as SHC (Bacteroidetes, Wilcoxon: *P* = 0.012; Firmicutes, Wilcoxon: *P* = 0.012) and CSC mice (Bacteroidetes, Wilcoxon: *P* = 0.016; Firmicutes, Wilcoxon: *P* = 0.016) of the *M. aurum* DSM 33539 group at days 19 vs. 1, respectively. This resulted in a significantly decreased F/B ratio (Figure 4C) in SHC mice of the Veh group (Wilcoxon: *P* = 0.008) as well as SHC (Wilcoxon: *P* = 0.020) and CSC mice (Wilcoxon: *P* = 0.016) of the *M. aurum* DSM 33539 group at days 19 vs. 1, respectively. Moreover, on day 19 the relative abundance of the phylum Desulfobacterota was significantly greater in Veh-treated CSC vs. SHC mice [Figure 4D; LMM: Factor *M. aurum* DSM 33539 × Factor Time × Factor Stress Interaction: *F*_(1, 27)_ = 12.31, *P* = 0.002; Factor Time: *F*_(1, 27)_ = 4.731, *P* = 0.039; Bonferroni: *P* = 0.008] and significantly reduced in CSC mice of the *M. aurum* DSM 33539 vs. Veh group (Bonferroni: *P* = 0.018). Furthermore, the relative abundance of the phylum Deferribacterota (Figure 4E) was reduced in both Veh (strong trend: Wilcoxon: *P* = 0.055) and *M. aurum* DSM 33539-treated (Wilcoxon: *P* = 0.043) SHC mice at day 19 vs. day 1, while the relative abundance of Deferribacterota was significantly increased in CSC vs. SHC mice of the Veh- (MWU: *P* = 0.030) but not *M. aurum* DSM 33539-treated group at day 19. Correlational analyses further revealed that the relative abundance of Deferribacterota on day 19 correlated positively with the histological damage score (Figure 4F; Spearman *r* = 0.48; Spearman *P* = 0.005) assessed after 8 days of DSS administration (i.e., day 28). The relative abundances of Patescibacteria, Proteobacteria, Cyanobacteria, Actinobacteriota, and Verrucomicrobiota (Figures 4G–K) were neither affected by CSC nor *M. aurum* DSM 33539.

## Discussion

The results of the present study indicate that repeated i.g. administrations of a heat-inactivated preparation of *M. aurum* DSM 33539 have stabilizing effects on the composition of the intestinal microbiome, indicated by an *M. aurum* DSM 33539-associated amelioration of the CSC-induced increase in the abundance of the colitogenic phyla Desulfobacterota and Deferribacterota. In line with the latter, *M. aurum* DSM 33539 was further protective against the aggravating effects of stress on subsequent DSS colitis, suggesting that the stress-protective effects reported for *M. vaccae* NCTC 11659 and *M. vaccae* ATCC 15483^T^ (Reber et al., 2016a; Amoroso et al., 2020; Langgartner et al., 2023; Amoroso et al., 2019; Loupy et al., 2021; Amoroso et al., 2021) are generalizable also to other closely related rapidly growing environmental saprophytic NTM species.

### *M. aurum* DSM 33539 protects against CSC-induced adrenal enlargement and aggravation of DSS-colitis

Adrenal enlargement has been shown to be the most predictive biomarker for classification and class prediction in the CSC paradigm (Langgartner et al., 2018) and a typical sign of chronic stress (Langgartner et al., 2015). In confirmation that the CSC paradigm worked reliably in the present study, Veh-treated CSC mice were characterized by larger adrenals compared with respective SHC mice. However, in contrast to our earlier studies showing that *M. vaccae* NCTC 11659 is not protective against CSC-induced hypothalamic-pituitary-adrenal (HPA) axis changes, including adrenal enlargement, neither when administered via the s.c. (Reber et al., 2016a; Mazzari et al., 2023) nor i.g. (Langgartner et al., 2023) route, CSC mice repeatedly administered i.g. with *M. aurum* DSM 33539 in the present study were not characterized by adrenal enlargement compared with respective SHC mice, at least when assessed following 7 days of DSS treatment initiated following the termination of the CSC paradigm. As repeated i.g. administrations of *M. aurum* DSM 33539 prior to CSC further had protective effects against the CSC-induced aggravation of DSS colitis (i.e., DSS in the drinking water between days 21 and 28) and as a local peripheral inflammation activates the HPA axis and, if chronic, promotes adrenal enlargement (Wolff et al., 2014), the protective effect of *M. aurum* DSM 33539 on CSC-induced adrenal enlargement might also reflect an indirect consequence of *M. aurum* DSM 33539 protecting against CSC-induced aggravation of DSS-induced colitis. The latter represents a widely used model of inflammatory bowel disease (IBD) (Reber et al., 2008; Reber, 2012), and the protective effect of *M. aurum* DSM 33539 is indicated by an increased body weight loss (i.e., days 21 until 28, days 24 until 28) and a higher histological damage score in CSC vs. SHC mice in the Veh but not in the *M. aurum* DSM 33539 group, while the colon length as a marker for inflammatory shortening of the colon was not affected at all, and the CSC-induced increase in *ex vivo* IFN-γ secretion from anti-CD3/CD28-stimulated mesenteric lymph node cells was comparable between Veh- and *M. aurum* DSM 33539-treated mice.

### *M. aurum* DSM 33539 stabilizes the composition of the intestinal microbiome

In an earlier study, we reported strong declines in α-diversity over time in both CSC and SHC mice administered (s.c.) repeatedly with BBS (i.e., vehicle for *M. vaccae* NCTC 11659), particularly evident at the onset of the CSC procedure (Reber et al., 2016a), suggesting that the CSC procedure is stressful for all mice, which are housed in the same room. A follow-up study suggests that dominant aggressor mice have been used that were positive for enterohepatic *Helicobacter* spp. (EHS) and that CSC but not SHC mice got infected with these EHS during CSC exposure (Langgartner et al., 2017). As CSC but not SHC mice of the BBS group further were characterized by an increased abundance of the phylum Proteobacteria, including the genus *Helicobacter* on days 8 and 15 of CSC exposure (Reber et al., 2016a), these data collectively support the hypothesis that a stress-induced decline in α-diversity facilitates the infection with and the expansion of certain colitogenic pathobionts, finally promoting stress-induced intestinal pathologies. Consistent with the latter, the abundance of both Proteobacteria and *Helicobacter* spp. correlated positively with the histological damage in the colon of SHC and CSC mice (Reber et al., 2016a). Moreover, other stress paradigms also have been shown to increase intestinal *Helicobacter* spp. abundance in mice (Guo et al., 2009), and intestinal *Helicobacter* abundance predicts intestinal inflammation scores in mice with impaired immunoregulation (IL-10^−/−^ mice), whereas unstressed wild-type (WT) mice do not develop intestinal inflammation in response to *Helicobacter* spp. infection (Solnick and Schauer, 2001; Bassett et al., 2015; Kullberg et al., 2001). Of note, in our earlier study assessing the stress-protective effects of repeated s.c. administrations of *M. vaccae* NCTC 11659 we further detected an effect of time for the abundance of the phylum Desulfobacterota, indexed by the expansion of an unidentified genus of Desulfovibrionaceae (Reber et al., 2016a). The phylum Desulfobacterota consists of many organisms that are involved in the degradation of butyrate (Gryaznova et al., 2022), suggesting their involvement in the stress-induced disruption of the intestinal barrier function (Voigt et al., 2022) and supporting the association between Desulfobacterota, an increased immune response and gut inflammation (Figliuolo et al., 2017; Tamargo et al., 2022; Rajput et al., 2023). As CSC but not SHC mice administered repeatedly (s.c.) with *M. vaccae* NCTC 11659 show a strong decline in α-diversity during the course of CSC (Reber et al., 2016a), these findings further suggest that *M. vaccae* NCTC 11659 is able to protect against the negative effects of mild witnessing stress on intestinal α-diversity in SHC, but not against the negative effects of severe chronic social defeat during CSC (Reber et al., 2016a). Again, the decline in intestinal α-diversity in *M. vaccae* NCTC 11659-administered CSC mice was paralleled by an increased abundance of Proteobacteria, including *Helicobacter* spp., on days 8 and 15 of CSC exposure.

In contrast to these earlier studies, the current study has been conducted under SPF conditions using dominant aggressor mice negative for EHS, with the consequence that CSC mice could not be infected with EHS during CSC exposure. The latter might explain why CSC vs. SHC mice of the Veh group in the present study were only characterized by an increased abundance of the phylum Desulfobacterota but not Proteobacteria on day 19 of CSC exposure. In line with the stabilizing effects reported earlier for repeated s.c. administration with *M. vaccae* NCTC 11659 on an unidentified genus of Desulfovibrionaceae (Reber et al., 2016a), repeated i.g. administration with *M. aurum* DSM 33539 in the present study prevented the CSC-induced expansion of Desulfobacterota, which might at least in part be mediating the stress-protective effects of *M. aurum* DSM 33539 on CSC-induced aggravation of DSS-induced colitis.

Interestingly, in the present study repeated *M. aurum* DSM 33539 administrations further prevented the CSC-induced increase in the relative abundance of Deferribacterota, which has been associated with exacerbated intestinal inflammation at steady state and following DSS treatment (Selvanantham et al., 2016). Noteworthy in this context is further that, among others, the abundances of both Desulfobacterota and Deferribacterota were decreased in mice supplemented with probiotic bacteria over several weeks (Gryaznova et al., 2022). Consistent with the latter, the abundance of Deferribacterota in the present study correlated positively with the histological damage in the colon of DSS-treated mice of all experimental groups.

Although a significant time effect for the Shannon diversity index, Faith's phylogenetic diversity and abundance of observed ASVs with lower values on days 19 vs. 1 of CSC argue for a general decline in α-diversity in all experimental groups housed in the same room, separate *t-*tests indicating a higher Shannon diversity index, Faith's phylogenetic diversity, and observed ASVs in CSC vs. SHC of the *M. aurum* DSM 33539 group on day 19 suggest that *M. aurum* DSM 33539 has at least mild protective effects against the decline in α-diversity between days 1 and 19 in CSC mice. Note, *M. vaccae* NCTC 11659 protected SHC from a witnessing stress-induced decline in α-diversity, but not CSC mice (Reber et al., 2016a).

We further revealed an increase in the abundance of Bacteroidetes in SHC mice of the Veh group between days 1 and 19 of CSC, while the abundance of Firmicutes and, consequently, the F/B ratio were declining. This is in line with what we reported earlier for SHC mice housed together with CSC mice in the same room (Reber et al., 2016a). As further chronic unpredictable immobilization stress as well as chronic social defeat stress have been shown to lower the fecal F/B ratio in male mice (Dodiya et al., 2020; Zhang et al., 2017), the current findings again support the conclusion that sensory contact to CSC mice represents at least a mild stressor for SHC mice able to affect the intestinal microbial composition. Noteworthy, statistical analysis revealed that CSC in contrast to SHC mice of the Veh group did not show the above-reported increase in the abundance of Bacteroidetes as well as a decrease in the abundance of Firmicutes and the F/B ratio between days 19 and 1, which is in line with our earlier study (Reber et al., 2016a) and indicating that CSC mice do not show any relative changes in these phyla or their ratio over time, which is probably due to the above-mentioned expansion of Desulfobacterota and Deferribacterota or Proteobacteria (Reber et al., 2016a), respectively. Consistent with the protective effects of *M. aurum* DSM 33539 against the CSC-induced increase in Desulfobacterota and Deferribacterota abundances in the present study, SHC and CSC mice of the *M. aurum* DSM 33539 group showed a higher abundance of Bacteroidetes and a decreased abundance of Firmicutes and F/B ratio at days 19 vs. 1.

### *M. aurum* DSM 33539 did not alter emotional behavior

In line with earlier studies conducted by our group investigating the stress-protective effects of *M. vaccae* NCTC 11659 (Amoroso et al., 2020, 2019), CSC mice repeatedly administered with sterile water as a vehicle for *M. aurum* DSM 33539 prior to stressor exposure did not reliably develop a behavioral phenotype characterized by increased general anxiety-related behavior. The latter might be due to the fact that most administration procedures, including s.c. (Amoroso et al., 2020), i.n. (Amoroso et al., 2019), and i.g. (present study) administrations, require immobilization of experimental mice and, thus, are *per se* stressful and anxiogenic, masking possible CSC effects on emotionality. In contrast, CSC in untreated mice reliably causes a behavioral phenotype characterized by increased anxiety-related behavior during elevated plus-maze, light–dark box, OF/NO, elevated platform, and SPAT exposure (Langgartner et al., 2015, 2017; Slattery et al., 2012; Reber et al., 2007, 2016b; Uschold-Schmidt et al., 2012). In support of the hypothesis that immobilization required for substance administrations *per se* represents a severe stressor resulting in increased anxiety levels and, thus, masking possible CSC effects on emotionality, acute restraint stress enhances anxiety-like behavior with a time-delay of ~10 days, measured as significant open arm avoidance in the EPM (Mitra et al., 2005) and accompanied by enhanced spine density on dendrites of basolateral amygdala neurons (BLA), a site for storage of fearful memories and/or stressful experiences (Blair et al., 2001; LeDoux, 1993; Rogan et al., 1997; Schafe et al., 2001). Given that prior administration of low-dose corticosterone was able to prevent these molecular and behavioral effects of acute restraint stress (Rao et al., 2012), administering Veh or *M. aurum* DSM 33539 during the dark phase, when corticosterone levels are elevated due to the diurnal rhythm, might be able to prevent the anxiogenic effects of the administration procedure *per se*. Of note, as repeated i.g. administrations of *M. aurum* DSM 33539 in the present study reduced the entries into closed arms during EPM testing in SHC mice as well as the distance moved in CSC mice during both OF and NO exploration, *M. aurum* DSM 33539 seems to decrease general locomotion, further impeding the interpretation of anxiogenic-like effects of *M. aurum* DSM 33539 in SHC mice, indicated by a decreased time spent on the OA during EPM testing, and CSC mice, indicated by an increased time in corners during NO exploration. However, as the distance moved during SPAT empty and social cage exploration was comparable between all experimental groups, the effects of *M. aurum* DSM 33539 on general locomotion seem to be rather mild and paradigm specific. Importantly, CSC-induced social anxiety seems to be less affected by repeated prior substance administrations than CSC-induced general anxiety, indicated by CSC in contrast to SHC mice of the Veh group not preferring the social over the empty cage. An effect of i.g. *M. aurum* DSM 33539 treatment on a CSC-induced lack of social preference could not be assessed, as *M. aurum* DSM 33539-treated SHC mice did not prefer the social over the empty cage.

## Conclusion

Collectively, the results of this study confirm and extend previous findings from our group and provide further support for the microbiome-stabilizing and stress-protective potential of rapidly growing environmental saprophytic NTM in a mouse model of chronic psychosocial stress.

## Data Availability

Code for reproducing microbiome analysis can be found at https://github.com/GRalexOSS/Maurum_Processing. The raw data supporting the conclusions of this article will be made available by the authors, without undue reservation.

## References

[B1] Amoroso M. Bottcher A. Lowry C. A. Langgartner D. Reber S. O. (2020). Subcutaneous *Mycobacterium vaccae* promotes resilience in a mouse model of chronic psychosocial stress when administered prior to or during psychosocial stress. Brain Behav. Immun. 87, 309–317. 10.1016/j.bbi.2019.12.018 31887415

[B2] Amoroso M. Kempter E. Eleslambouly T. Lowry C. A. Langgartner D. Reber S. O.. (2019). Intranasal *Mycobacterium vaccae* administration prevents stress-induced aggravation of dextran sulfate sodium (DSS) colitis. Brain Behav. Immun. 80, 595–604. 10.1016/j.bbi.2019.05.005 31059809

[B3] Amoroso M. Langgartner D. Lowry C. A. Reber S. O. (2021). Rapidly growing *Mycobacterium* species: the long and winding road from tuberculosis vaccines to potent stress-resilience agents. Int. J. Mol. Sci. 22:12938. 10.3390/ijms222312938 34884743 PMC8657684

[B4] Appiah S. A. Foxx C. L. Langgartner D. Palmer A. Zambrano C. A. Braumuller S.. (2021). Evaluation of the gut microbiome in association with biological signatures of inflammation in murine polytrauma and shock. Sci. Rep. 11:6665. 10.1038/s41598-021-85897-w 33758228 PMC7988149

[B5] Bassett S. A. Young W. Barnett M. P. Cookson A. L. McNabb W. C. Roy N. C.. (2015). Changes in composition of caecal microbiota associated with increased colon inflammation in interleukin-10 gene-deficient mice inoculated with *Enterococcu*s species. Nutrients 7, 1798–1816. 10.3390/nu7031798 25768951 PMC4377882

[B6] Blair H. T. Schafe G. E. Bauer E. P. Rodrigues S. M. LeDoux J. E. (2001). Synaptic plasticity in the lateral amygdala: a cellular hypothesis of fear conditioning. Learn Mem. 8, 229–242. 10.1101/lm.30901 11584069

[B7] Bolyen E. Rideout J. R. Dillon M. R. Bokulich N. A. Abnet C. C. Al-Ghalith G. A.. (2019). Reproducible, interactive, scalable and extensible microbiome data science using QIIME 2. Nat. Biotechnol. 37, 852–857. 10.1038/s41587-019-0209-9 31341288 PMC7015180

[B8] Bowers S. J. Lambert S. He S. Lowry C. A. Fleshner M. Wright K. P.. (2020). Immunization with a heat-killed bacterium, *Mycobacterium vaccae* NCTC 11659, prevents the development of cortical hyperarousal and a PTSD-like sleep phenotype after sleep disruption and acute stress in mice. Sleep 44:zsaa271. 10.1101/2020.05.07.082859 33283862 PMC8193553

[B9] Callahan B. J. McMurdie P. J. Rosen M. J. Han A. W. Johnson A. J. Holmes S. P.. (2016). DADA2: high-resolution sample inference from Illumina amplicon data. Nat. Methods 13, 581–583. 10.1038/nmeth.3869 27214047 PMC4927377

[B10] Dodiya H. B. Forsyth C. B. Voigt R. M. Engen P. A. Patel J. Shaikh M.. (2020). Chronic stress-induced gut dysfunction exacerbates Parkinson's disease phenotype and pathology in a rotenone-induced mouse model of Parkinson's disease. Neurobiol. Dis. 135:104352. 10.1016/j.nbd.2018.12.012 30579705

[B11] Figliuolo V. R. dos Santos L. M. Abalo A. Nanini H. Santos A. Brittes N. M.. (2017). Sulfate-reducing bacteria stimulate gut immune responses and contribute to inflammation in experimental colitis. Life Sci. 189, 29–38. 10.1016/j.lfs.2017.09.014 28912045

[B12] Foertsch S. Reber S. O. (2020). The role of physical trauma in social stress-induced immune activation. Neurosci. Biobehav. Rev. 113, 169–178. 10.1016/j.neubiorev.2020.02.025 32109454

[B13] Fonken L. K. Frank M. G. D'Angelo H. M. Heinze J. D. Watkins L. R. Lowry C. A.. (2018b). *Mycobacterium vaccae* immunization protects aged rats from surgery-elicited neuroinflammation and cognitive dysfunction. Neurobiol. Aging 71, 105–114. 10.1016/j.neurobiolaging.2018.07.012 30118926 PMC6162105

[B14] Fonken L. K. Frank M. G. Gaudet A. D. Maier S. F. (2018a). Stress and aging act through common mechanisms to elicit neuroinflammatory priming. Brain Behav. Immun. 73, 133–148. 10.1016/j.bbi.2018.07.012 30009999 PMC6129421

[B15] Foxx C. L. Heinze J. D. Gonzalez A. Vargas F. Baratta M. V. Elsayed A. I.. (2020). Effects of immunization with the soil-derived bacterium *Mycobacterium vaccae* on stress coping behaviors and cognitive performance in a “two hit” stressor model. Front. Physiol. 11:524833. 10.3389/fphys.2020.524833 33469429 PMC7813891

[B16] Frank M. G. Fonken L. K. Dolzani S. D. Annis J. L. Siebler P. H. Schmidt D.. (2018). Immunization with *Mycobacterium vaccae* induces an anti-inflammatory milieu in the CNS: Attenuation of stress-induced microglial priming, alarmins and anxiety-like behavior. Brain Behav. Immun. 73, 352–363. 10.1016/j.bbi.2018.05.020 29807129 PMC6129419

[B17] Frank M. G. Fonken L. K. Watkins L. R. Maier S. F. Lowry C. A. (2019). Could probiotics be used to mitigate neuroinflammation? ACS Chem. Neurosci. 10, 13–15. 10.1021/acschemneuro.8b00386 30109920

[B18] Gola H. Engler H. Sommershof A. Adenauer H. Kolassa S. Schedlowski M.. (2013). Posttraumatic stress disorder is associated with an enhanced spontaneous production of pro-inflammatory cytokines by peripheral blood mononuclear cells. BMC Psychiatry 13:40. 10.1186/1471-244X-13-40 23360282 PMC3574862

[B19] Grubbs F. E. (1969). Procedures for detecting outlying observations in samples. Technometrics 11, 1–21. 10.1080/00401706.1969.10490657

[B20] Gryaznova M. Dvoretskaya Y. Burakova I. Syromyatnikov M. Popov E. Kokina A.. (2022). Dynamics of changes in the gut microbiota of healthy mice fed with lactic acid bacteria and bifidobacteria. Microorganisms 10:1020. 10.3390/microorganisms10051020 35630460 PMC9144108

[B21] Guo G. Jia K. R. Shi Y. Liu X. F. Liu K. Y. Qi W.. (2009). Psychological stress enhances the colonization of the stomach by *Helicobacter pylori* in the BALB/c mouse. Stress 12, 478–485. 10.3109/10253890802642188 20102319

[B22] Hassell J. E. Jr. Fox J. H. Arnold M. R. Siebler P. H. Lieb M. W. Schmidt D.. (2019). Treatment with a heat-killed preparation of *Mycobacterium vaccae* after fear conditioning enhances fear extinction in the fear-potentiated startle paradigm. Brain Behav. Immun. 81, 151–160. 10.1016/j.bbi.2019.06.008 31175996 PMC6754802

[B23] Hodes G. E. Pfau M. L. Leboeuf M. Golden S. A. Christoffel D. J. Bregman D.. (2014). Individual differences in the peripheral immune system promote resilience versus susceptibility to social stress. Proc. Natl. Acad. Sci. U. S. A. 111, 16136–16141. 10.1073/pnas.1415191111 25331895 PMC4234602

[B24] Janssen S. McDonald D. Gonzalez A. Navas-Molina J. A. Jiang L. Xu Z. Z.. (2018). Phylogenetic placement of exact amplicon sequences improves associations with clinical information. mSystems 3:e00021-18. 10.1128/mSystems.00021-18 29719869 PMC5904434

[B25] Kempter E. Amoroso M. Duffner H. L. Werner A. M. Langgartner D. Kupfer S.. (2021). Changes in functional glucocorticoid sensitivity of isolated splenocytes induced by chronic psychosocial stress – a time course study. Front. Immunol. 12:753822. 10.3389/fimmu.2021.753822 34675935 PMC8523951

[B26] Kempter E. Amoroso M. Kupfer S. Lupu L. Kustermann M. Scheurer J.. (2023). The PMN-MDSC – A key player in glucocorticoid resistance following combined physical and psychosocial trauma. Brain Behav. Immun. 108, 148–161. 10.1016/j.bbi.2022.11.011 36427809

[B27] Kivimaki M. Shipley M. J. Batty G. D. Hamer M. Akbaraly T. N. Kumari M.. (2014). Long-term inflammation increases risk of common mental disorder: a cohort study. Mol. Psychiatry 19, 149–150. 10.1038/mp.2013.35 23568195 PMC3903110

[B28] Kullberg M. C. Rothfuchs A. G. Jankovic D. Caspar P. Wynn T. A. Gorelick P. L.. (2001). *Helicobacter hepaticus*-induced colitis in interleukin-10-deficient mice: cytokine requirements for the induction and maintenance of intestinal inflammation. Infect. Immun. 69, 4232–4241. 10.1128/IAI.69.7.4232-4241.2001 11401959 PMC98456

[B29] Langgartner D. Amoroso M. Kempter E. Kustermann M. Scheurer J. Lowry C. A.. (2023). *Mycobacterium vaccae* protects against glucocorticoid resistance resulting from combined physical and psychosocial trauma in mice. Brain Behav. Immun. 109, 221–234. 10.1016/j.bbi.2023.01.018 36736929

[B30] Langgartner D. Füchsl A. M. Kaiser L. M. Meier T. Foertsch S. Buske C.. (2018). Biomarkers for classification and class prediction of stress in a murine model of chronic subordination stress. PLoS ONE 13:e0202471. 10.1371/journal.pone.0202471 30183738 PMC6124755

[B31] Langgartner D. Füchsl A. M. Uschold-Schmidt N. Slattery D. A. Reber S. O. (2015). Chronic subordinate colony housing paradigm: a mouse model to characterize the consequences of insufficient glucocorticoid signaling. Front. Psychiatry 6:18. 10.3389/fpsyt.2015.00018 25755645 PMC4337237

[B32] Langgartner D. Lowry C. A. Reber S. O. (2019). Old Friends, immunoregulation, and stress resilience. Pflügers Archiv. 471, 237–269. 10.1007/s00424-018-2228-7 30386921 PMC6334733

[B33] Langgartner D. Peterlik D. Foertsch S. Füchsl A. M. Brokmann P. Flor P. J.. (2017). Individual differences in stress vulnerability: the role of gut pathobionts in stress-induced colitis. Brain Behav. Immun. 64, 23–32. 10.1016/j.bbi.2016.12.019 28012830

[B34] LeDoux J. E. (1993). Emotional memory systems in the brain. Behav. Brain Res. 58, 69–79. 10.1016/0166-4328(93)90091-4 8136051

[B35] Loupy K. M. Cler K. E. Marquart B. M. Yifru T. W. D'Angelo H. M. Arnold M. R.. (2021). Comparing the effects of two different strains of *Mycobacterium vaccae, M. vaccae* NCTC 11659 and *M. vaccae* ATCC 15483, on stress-resilient behaviors and lipid-immune signaling in rats. Brain Behav. Immun. 91, 212–229. 10.1016/j.bbi.2020.09.030 33011306 PMC7749860

[B36] Martínez I. James S. C. Maldonado-Gómez M. Eren A. M. Siba P. M. Greenhill A. R.. (2015). The gut microbiota of rural Papua New Guineans: composition, diversity patterns, and ecological processes. Cell Rep. 11, 527–538. 10.1016/j.celrep.2015.03.049 25892234

[B37] Mazzari G. Lowry C. A. Langgartner D. Reber S. O. (2023). Subcutaneous *Mycobacterium vaccae* ameliorates the effects of early life adversity alone or in combination with chronic stress during adulthood in male and female mice. Neurobiol. Stress 26:100568. 10.1016/j.ynstr.2023.100568 37727147 PMC10506060

[B38] Mitra R. Jadhav S. McEwen B. S. Vyas A. Chattarji S. (2005). Stress duration modulates the spatiotemporal patterns of spine formation in the basolateral amygdala. Proc. Natl. Acad. Sci. U. S. A. 102, 9371–9376. 10.1073/pnas.0504011102 15967994 PMC1166638

[B39] Nouioui I. Dye T. (2021). Heat-killed *Mycolicibacterium aurum Aogashima*: an environmental nonpathogenic actinobacteria under development as a safe novel food ingredient. Food Sci. Nutr. 9, 4839–4854. 10.1002/fsn3.2413 34531996 PMC8441333

[B40] Pace T. W. Mletzko T. C. Alagbe O. Musselman D. L. Nemeroff C. B. Miller A. H.. (2006). Increased stress-induced inflammatory responses in male patients with major depression and increased early life stress. Am. J. Psychiatry 163, 1630–1633. 10.1176/ajp.2006.163.9.1630 16946190

[B41] Peen J. Schoevers R. A. Beekman A. T. Dekker J. (2010). The current status of urban-rural differences in psychiatric disorders. Acta Psychiatr. Scand. 121, 84–93. 10.1111/j.1600-0447.2009.01438.x 19624573

[B42] Percie du Sert N. Ahluwalia A. Alam S. Avey M. T. Baker M. Browne W. J.. (2020). Reporting animal research: explanation and elaboration for the ARRIVE guidelines 2.0. PLoS Biol. 18:e3000411. 10.1371/journal.pbio.3000411 32663221 PMC7360025

[B43] Quast C. Pruesse E. Yilmaz P. Gerken J. Schweer T. Yarza P.. (2013). The SILVA ribosomal RNA gene database project: improved data processing and web-based tools. Nucl. Acids Res. 41(Database issue), D590–D596. 10.1093/nar/gks1219 23193283 PMC3531112

[B44] Rajput M. Momin T. Singh A. Banerjee S. Villasenor A. Sheldon J.. (2023). Determining the association between gut microbiota and its metabolites with higher intestinal immunoglobulin A response. Vet. Anim. Sci. 19:100279. 10.1016/j.vas.2022.100279 36533218 PMC9755367

[B45] Rao R. P. Anilkumar S. McEwen B. S. Chattarji S. (2012). Glucocorticoids protect against the delayed behavioral and cellular effects of acute stress on the amygdala. Biol. Psychiatry 72, 466–475. 10.1016/j.biopsych.2012.04.008 22572034 PMC3753225

[B46] Reber S. O. (2012). Stress and animal models of inflammatory bowel disease—An update on the role of the hypothalamo–pituitary–adrenal axis. Psychoneuroendocrinology 37, 1–19. 10.1016/j.psyneuen.2011.05.014 21741177

[B47] Reber S. O. Birkeneder L. Veenema A. H. Obermeier F. Falk W. Straub R. H.. (2007). Adrenal insufficiency and colonic inflammation after a novel chronic psycho-social stress paradigm in mice: implications and mechanisms. Endocrinology 148, 670–682. 10.1210/en.2006-0983 17110427

[B48] Reber S. O. Langgartner D. Foertsch S. Postolache T. T. Brenner L. A. Guendel H.. (2016b). Chronic subordinate colony housing paradigm: a mouse model for mechanisms of PTSD vulnerability, targeted prevention, and treatment-−2016 Curt Richter Award Paper. Psychoneuroendocrinology 74, 221–230. 10.1016/j.psyneuen.2016.08.031 27676359

[B49] Reber S. O. Obermeier F. Straub R. H. Veenema A. H. Neumann I. D. (2008). Aggravation of DSS-induced colitis after chronic subordinate colony (CSC) housing is partially mediated by adrenal mechanisms. Stress 11, 225–234. 10.1080/10253890701733351 18465469

[B50] Reber S. O. Peters S. Slattery D. A. Hofmann C. Schölmerich J. Neumann I. D.. (2011). Mucosal immunosuppression and epithelial barrier defects are key events in murine psychosocial stress-induced colitis. Brain Behav. Immun. 25, 1153–1161. 10.1016/j.bbi.2011.03.004 21397685

[B51] Reber S. O. Siebler P. H. Donner N. C. Morton J. T. Smith D. G. Kopelman J. M.. (2016a). Immunization with a heat-killed preparation of the environmental bacterium *Mycobacterium vaccae* promotes stress resilience in mice. Proc. Natl. Acad. Sci. U. S. A. 113, E3130–E3139. 10.1073/pnas.1600324113 27185913 PMC4896712

[B52] Riedler J. Braun-Fahrländer C. Eder W. Schreuer M. Waser M. Maisch S.. (2001). Exposure to farming in early life and development of asthma and allergy: a cross-sectional survey. Lancet. 358, 1129–1133. 10.1016/S0140-6736(01)06252-3 11597666

[B53] Rogan M. T. Staubli U. V. LeDoux J. E. (1997). Fear conditioning induces associative long-term potentiation in the amygdala. Nature 390, 604–607. 10.1038/37601 9403688

[B54] Rohleder N. (2014). Stimulation of systemic low-grade inflammation by psychosocial stress. Psychosom. Med. 76, 181–189. 10.1097/PSY.0000000000000049 24608036

[B55] Rook G. A. (2013). Regulation of the immune system by biodiversity from the natural environment: an ecosystem service essential to health. Proc. Natl. Acad. Sci. U. S. A. 110, 18360–18367. 10.1073/pnas.1313731110 24154724 PMC3831972

[B56] Rook G. A. Raison C. L. Lowry C. A. (2013). Childhood microbial experience, immunoregulation, inflammation and adult susceptibility to psychosocial stressors and depression in rich and poor countries. Evol. Med. Public Health 2013, 14–17. 10.1093/emph/eos005 24481181 PMC4183960

[B57] Sanchez K. Darling J. S. Kakkar R. Wu S. L. Zentay A. Lowry C. A.. (2022). *Mycobacterium vaccae* immunization in rats ameliorates features of age-associated microglia activation in the amygdala and hippocampus. Sci. Rep. 12:2165. 10.1038/s41598-022-05275-y 35140249 PMC8828872

[B58] Schafe G. E. Nader K. Blair H. T. LeDoux J. E. (2001). Memory consolidation of Pavlovian fear conditioning: a cellular and molecular perspective. Trends Neurosci. 24, 540–546. 10.1016/S0166-2236(00)01969-X 11506888

[B59] Selvanantham T. Lin Q. Guo C. X. Surendra A. Fieve S. Escalante N. K.. (2016). NKT cell-deficient mice harbor an altered microbiota that fuels intestinal inflammation during chemically induced colitis. J. Immunol. 197, 4464–4472. 10.4049/jimmunol.1601410 27799307

[B60] Singewald G. M. Nguyen N. K. Neumann I. D. Singewald N. Reber S. O. (2009). Effect of chronic psychosocial stress-induced by subordinate colony (CSC) housing on brain neuronal activity patterns in mice. Stress 12, 58–69. 10.1080/10253890802042082 19116889

[B61] Slattery D. A. Uschold N. Magoni M. Bär J. Popoli M. Neumann I. D.. (2012). Behavioural consequences of two chronic psychosocial stress paradigms: anxiety without depression. Psychoneuroendocrinology 37, 702–714. 10.1016/j.psyneuen.2011.09.002 21962377

[B62] Solnick J. V. Schauer D. B. (2001). Emergence of diverse *Helicobacter* species in the pathogenesis of gastric and enterohepatic diseases. Clin. Microbiol. Rev. 14, 59–97. 10.1128/CMR.14.1.59-97.2001 11148003 PMC88962

[B63] Tamargo A. Molinero N. Reinosa J. J. Alcolea-Rodriguez V. Portela R. Bañares M. A.. (2022). PET microplastics affect human gut microbiota communities during simulated gastrointestinal digestion, first evidence of plausible polymer biodegradation during human digestion. Sci. Rep. 12:528. 10.1038/s41598-021-04489-w 35017590 PMC8752627

[B64] United Nations Department of Economic and Social Affairs, Population Division. (2014). World Urbanization Prospects: The 2014 Revision, Highlights (ST/ESA/SER.A/352). New York, NY.

[B65] Uschold-Schmidt N. Nyuyki K. D. Fuchsl A. M. Neumann I. D. Reber S. O. (2012). Chronic psychosocial stress results in sensitization of the HPA axis to acute heterotypic stressors despite a reduction of adrenal in vitro ACTH responsiveness. Psychoneuroendocrinology 37, 1676–1687. 10.1016/j.psyneuen.2012.02.015 22444976

[B66] Voigt R. M. Zalta A. K. Raeisi S. Zhang L. Brown J. M. Forsyth C. B.. (2022). Abnormal intestinal milieu in posttraumatic stress disorder is not impacted by treatment that improves symptoms. Am. J. Physiol. Gastrointest. Liver Physiol. 323, G61–G70. 10.1152/ajpgi.00066.2022 35638693 PMC9291416

[B67] Weiss S. Xu Z. Z. Peddada S. Amir A. Bittinger K. Gonzalez A.. (2017). Normalization and microbial differential abundance strategies depend upon data characteristics. Microbiome 5, 1–18. 10.1186/s40168-017-0237-y 28253908 PMC5335496

[B68] Wolff C. Krinner K. Schroeder J. A. Straub R. H. (2014). Inadequate corticosterone levels relative to arthritic inflammation are accompanied by altered mitochondria/cholesterol breakdown in adrenal cortex: a steroid-inhibiting role of IL-1beta in rats. Ann. Rheum. Dis. 74, 1890–1897. 10.1136/annrheumdis-2013-203885 24812291

[B69] Zhang J. C. Yao W. Dong C. Yang C. Ren Q. Ma M.. (2017). Blockade of interleukin-6 receptor in the periphery promotes rapid and sustained antidepressant actions: a possible role of gut-microbiota-brain axis. Transl. Psychiatry 7:e1138. 10.1038/tp.2017.112 28556833 PMC5534942

